# Do Platelet-Rich Plasma Injections for Knee Osteoarthritis Work?

**DOI:** 10.7759/cureus.34533

**Published:** 2023-02-02

**Authors:** Abbas Shahid, Asad Malik, Ali Bukhari, Adil Shaikh, Jill Rutherford, Bilal Barkatali

**Affiliations:** 1 Trauma & Orthopedics, Salford Royal NHS Foundation Trust, Manchester, GBR; 2 Emergency Medicine, Warrington and Halton Hospitals NHS Trust, Manchester, GBR; 3 Emergency Medicine, University Hospitals of North Midlands NHS Trust, Manchester, GBR; 4 Vascular Surgery, Northern Care Alliance NHS Group, Manchester, GBR

**Keywords:** platelet-rich plasma/prp, knee osteoarthritis/koa, knee oa, chronic pain management, orthopaedics surgery, platelet-rich plasma therapy for joints, knee osteo-arthritis

## Abstract

Background: Recent evidence suggests a benefit in platelet-rich plasma injections (PRP) for the knee in the management of mild to moderate osteoarthritis (OA). There is a reported reduction in pain, stiffness, and improved function. However, there is very little level-one literature available that supports this practice and conclusively proves a benefit gained throughout the course. Three main randomized control trials (RCTs) conducted in North America are often referenced and cited to prove their efficacy. This study aimed to look at the outcomes of patients having undergone this treatment to determine if there was any benefit.

Aims: This study aimed to determine if PRP injections administered in patients with knee OA over a six to eight-week time period demonstrated any benefit.

Methods: The Western Ontario and McMaster Universities arthritis index (WOMAC) tool was used before each of the three PRP injections over the six to eight-week period, and six weeks after the final injection in 31 patients. Each injection was given spaced two to three weeks apart. The outcomes observed were pain, stiffness, and physical function, and the total WOMAC score was calculated.

Results: The third injection showed a reduction in total WOMAC score, pain, stiffness, and physical function by 16.36%, 16.37%, 5.12%, and 18.03%, respectively. However, all scores returned close to baseline at the sixth-week follow-up post treatment.

Conclusion: Results showed a trend of reduction in the WOMAC score. However, they are overall indicative of a placebo effect from the injections. Further studies are needed to explore whether the grade of OA and patients’ weight have a significant impact on the results.

## Introduction

Osteoarthritis (OA) over time has become an increasingly prevalent condition as a result of the increasing life expectancy with modern medicine. Affecting the joints in our bodies, OA is the main condition resulting in disability across the globe. The Framingham osteoarthritis study [1], reported data demonstrating that OA was on equal footing with cardiovascular and respiratory conditions such as chronic obstructive pulmonary disease, and is as dominant by causing long-term physical disabilities with numerous studies reporting figures of approximately a quarter of a billion people affected globally [2-4].

Although the true pathophysiology of OA is not well understood, many definitions exist. For this study, we used the definition for osteoarthritis commonly cited as, *characteristic structural alterations of the joint, including focal degradation of articular cartilage and remodeling of subchondral bone with the formation of osteophytes at the joint margins, as well as an illness defined by a person’s symptoms, including pain, fatigue, mood alterations, and sleep disturbance* [2,5].

Knee osteoarthritis (KOA) is further classically defined using the Kellgren-Lawrence system, a radiological classification system that has been in use for around four decades, which on a scale from 1 to 4 has 2, 3, and 4 being mild, moderate, and severe, respectively [3,6]. Osteoarthritis commonly affects the knee accounting for 83% of all OA with the disease burden expected to rise exponentially [3]. Kurtz et al. suggest figures as high as a 673% increase in demand for operative procedures in the USA by 2030 [7].

In the United Kingdom, there are clear National Institute for Health and Care Excellence (NICE) guidelines that are utilized commonly in the management of KOA. These include addressing modifiable and non-modifiable risk factors including weight loss and lifestyle modification. They also include multi-modal therapies including analgesics, injections, and ultimately when conservative management has failed, surgical options [8]. All treatments have been met with differing levels of benefit for patients [9,10]. By far the measure that is of the greatest benefit for the treatment of KOA is exercise and improving general health [11-13]. In those patients with OA who are obese, this change is very successful in reducing pain, which is a key complaint in KOA [14].

Amongst recommended analgesics are non-steroidal anti-inflammatory drugs (NSAID) which are very effective in reducing pain. However, the consequences related to long-term NSAID use, particularly in the elderly, could likely overshadow possible benefits that are gained in attempting to manage KOA [10,15]. Alternative long-term therapeutic management options are available in the form of intra-articular (IA) injections. Injection of steroids has proven to be contentious at best and has mixed results with some patients reporting short-term benefits. However, all steroid preparations are known to be chondrotoxic with frequent use [6]. Hyaluronic acid (HA) is not supported by NICE in the United Kingdom but is frequently used in the USA although it is expensive to manufacture [16]. A recently commonly used tool for management, in addition to the ones discussed, is platelet-rich plasma (PRP) IA injections. This provides delivery of autologous growth factors which aid tissue repair [6]. Patients who receive PRP injections are also told to stop NSAIDs during the course. Previous studies such as the one conducted by Schippinger et al. discuss how NSAIDs impair platelet function in patients who receive PRP injections [17]. The reduction of cyclooxygenase-mediated oxygen consumption affects platelet aggregation resulting in the reduction in the release of bioactive agents such as growth factors. This would therefore reduce the efficacy of the PRP injections if used concurrently [17].

Currently, NICE has produced the following statement on the use of PRP in KOA, *although the interventional procedures advisory committee has not identified specific audit standards for the data collected, clinicians are advised to review their practice against the outcomes of this procedure* [18].

Three main double-blind randomized control trials (RCTs) are cited above, discussing the evidence for PRP injections in the knee [6,16,19]. These studies were conducted with patients receiving the PRP injections and compared to controls of normal saline or hyaluronic acid to determine if there is a therapeutic benefit. Three primary outcomes were observed, namely pain, stiffness, and physical function of the joint, using the extensive WOMAC questionnaire and scoring system. These three RCTs demonstrated a benefit in all three domains. The PRP injections are most commonly performed in patients with a Kellgren-Lawrence grade 2 or 3 osteoarthritis which translates to mild or moderate osteoarthritis in patients’ knees as these are the groups that are found to have the best relief [6,16,19].

Aims

This study aimed to determine if PRP injections administered In patients with KOA over a six to eight-week time period demonstrated any benefit. Similarly, using the WOMAC questionnaire (see Appendices), outcomes were investigated to determine whether there is a clinical benefit to the patient, a placebo effect, or if there is no value in them. These analyses were done similarly to the three RCTs discussed above [6,16,19] by individually investigating total score, pain, stiffness, and physical function. Comments from patients after the course of three injections were also analyzed to determine if there are any subjective benefits.

## Materials and methods

This is a retrospective study with data obtained from two consultants who performed the PRP injections in a similar and therefore comparable way. In total, 31 patients were included in the study. These procedures were performed at three main hospitals in Great Britain: Salford Royal hospital, Trafford general hospital, and Oaklands hospital. All patients routinely completed the WOMAC questionnaire on attendance.

In total, three injections were performed within a six to eight-week period with each patient having approximately, two to three weeks between injections. The WOMAC questionnaires were taken at each visit before the injection had taken place. The patients were followed up six weeks after their last injection with another WOMAC questionnaire undertaken at that point and a final comment provided. This was to ascertain if there was any subjective benefit to the patient.

The PRP injection was performed in a standardized manner and injected into the IA surface of the affected knee joint. Around 15ml of blood was taken from the antecubital fossa of each patient. This was spun in a centrifuge at 1500rpm for a total of five minutes. The Arthrex autologous chondrocyte plasma (ACP) double syringe system (Arthrex Inc., Naples, FL, USA) was utilized in the same method as in the Smith study [6]. All patients’ knees were prepped with chlorhexidine and sterility was maintained with dressings applied after the procedure. For the purposes of this study, the WOMAC questionnaire tool was utilized in order to characterize the key outcomes in patients with KOA. The WOMAC questionnaire is a standardized tool used to characterize three main complications associated with KOA: pain (20), stiffness (8), and physical functions (68). The results of these three categories are summed up to give a total WOMAC score out of 96. This is a tool often used and discussed in the level-one studies discussed above. In this study, the key difference was our questionnaire that had pain set on a 28-point scale. Two extra questions were present asking about the pain in each knee on a four-point scale of none, mild, moderate, and severe. Each of the three categories and total WOMAC scores was assessed and compared throughout the four time points at which the data was collected. Each none, mild, moderate, severe, and extreme were awarded 0 to 4, respectively, with totals for each category and an overall score out of 104 calculated.

In order to perform data analysis, statistical tests were carried out to determine if there was any statistical significance between the four time points at which data was collected. Data was input into the GraphPad Prism 7 software (Dotmatics, Boston, MA, USA) in order to carry this out. Basic statistics were carried out on each group with results shown as mean and standard deviation. The data was then found to be non-parametric so a Kruskal-Wallis test with Dunns multiple comparisons between time points was conducted as opposed to the ANOVA. The p-value significance level was set at 0.05 for all tests conducted. Results were all tabulated with the percentage change from baseline calculated to clearly demonstrate any change. Data was collected from the Electronic patients' record (EPR) system at Salford Royal Hospital and the Oaklands Hospital patient notes with X-rays reviewed on the X-ray viewing software at both hospitals.

## Results

A large majority of patients in this study (as shown in Table 1) had OA grades 2 and 3 and were overweight, with the overwhelming bulk diagnosed as having grade 3 OA. The yield of PRP that was injected into each patient differed slightly with the range between 3.5ml to 6ml. The average yield injected was 4.96ml for each PRP injection that was provided. 

**Table 1:** Summary table of demographics for patients included in this study OA: Osteoarthritis

Demographic details		Mean	% of patients (n=31)
Age		56.4	
Sex	Female		55%
	Male		45%
Knee	Left		42%
	Right		58%
OA Kellgren-Lawrence classification	1		0%
	2		16.7%
	3		80%
	4		3.3%
Weight	Slim build (BMI < 18.5)		10.3%
	Moderate build (BMI 18.5 to 24.9)		27.6%
	Overweight (BMI 25.0 to 29.9)		38%
	Very overweight (BMI > 30.0)		24.1%

The results in table 2 show that there is a reduction in scores from the baseline between each category. This is followed by a rise at the six-week follow-up post the third injection which is extremely close to baseline values. None of these changes from baseline until six weeks post treatment was statistically significant upon Kruskal-Wallis with Dunn's multiple comparisons tests. Total WOMAC score, stiffness, and physical function showed the largest improvements with a reduction in scores before the third injection administration. These rose close to baseline at the six-week follow-up after treatment. 

**Table 2:** Summary of change in outcomes throughout the follow-up WOMAC scores for all patients WOMAC: Western Ontario and McMaster Universities arthritis index All data presented with mean ± standard deviation

	Baseline	Before 2^nd^ injection	Before 3^rd^ injection	Six weeks following the 3^rd^ injection
Total	55.63 (± 16.51)	49.90 (± 15.62)	47.21 (± 18.21)	54 (± 16.08)
% change from baseline		-10.30%	-15.14%	-2.93%
Pain	14.08 (± 4.91)	12.50 (± 4.63)	11.95 (± 4.50)	14.57 (± 5.35)
% change from baseline		--11.22%	-15.13%	+3.48%
Stiffness	5.21 (± 1.47)	4.85 (± 1.39)	4.95 (± 1.51)	4.86 (± 1.57)
% change from baseline		-6.91%	-4.99%	-6.72%
Physical function	36.33 (± 11.85)	32.55 (± 11.10)	30.32 (± 13.68)	34.57 (± 10.39)
% change from baseline		-10.40%	-16.54%	-4.84%

As shown in Table 3, we observed a reduction in all scores up until before the third injection was given. However, all scores rose above baseline at follow-up after treatment had been completed. Similarly, none of these changes from baseline until the follow-up six weeks after treatment were statistically significant upon Kruskal-Wallis with Dunn's multiple comparisons tests. In comparison to the total group, the greatest improvements were seen in patients with mild OA whilst treatment was being undertaken. However, patients in this subset had higher scores after treatment in comparison to the results shown in Table 2. In total, only five patients were in the mild OA cohort.

**Table 3:** Summary of change in outcomes throughout follow-up of the WOMAC score for all patients with mild OA OA: Osteoarthritis, WOMAC: Western Ontario and McMaster Universities arthritis index All data presented with mean ± standard deviation

Mild OA Group	Baseline	Before 2^nd^ injection	Before 3^rd^ injection	Six weeks following the 3^rd^ injection
Total	54.33 (± 19.66)	46.55 (± 13.44)	35.50 (± 13.18)	61.00 (± 5.66)
% change from baseline		-14.32%	-34.66	+12.28%
Pain	14.00 (± 4.00)	12.50 (± 0.71)	11.25 (± 3.78)	17.00 (± 1.41)
% change from baseline		-10.71%	-19.64	+21.43%
Stiffness	4.67 (± 1.16)	3.50 (± 0.71)	3.75 (± 1.26)	6.50 (± 0.71)
% change from baseline		-25.05%	-19.70%	+39.19%
Physical function	35.67 (± 15.32)	30.50 (± 12.02)	20.50 (± 8.81)	37.50 (± 4.95)
% change from baseline		-14.49%	-42.53%	+5.13%

In total, six patients with moderate and three with mild OA, respectively, provided comments. A large majority of the comments patients provided as shown in Table 4 were that the injections were not of any benefit, and for those that it was beneficial, they said it only helped a little bit.

**Table 4:** Table showing comments provided by patients at their six-week follow-up after their course of three injections OA: Osteoarthritis

Patient comments post following the full course of injections	OA grade
No benefit	Mild
Has worked to some degree and can walk, and has reduced the amount of painkillers	Moderate
Increased anterior knee pain after 1st injection knee pain on driving; after the third injection, was pain-free for two weeks and overall has had only marginal improvement	Moderate
Has definitely relived the symptoms that I was experiencing and is much better than before	Mild
Only had 1 week's worth of benefits, now back to the course it was before	Moderate
Probably helped it about 50%; better but still in pain. But overall was definitely beneficial to do, just didn’t completely get rid of the symptoms.	Moderate
No benefit	Moderate
No benefit	Moderate
Has not made that much of a significant improvement	Mild

## Discussion

In this study, it was found that although PRP injections in the knee do demonstrate reductions in all modalities assessed, there was no statistically significant change. Over our study period in total, 12 to 14 weeks elapsed from the start of treatment until follow-up. Therefore, the results from this study to others will be compared in a similar time frame. Results from two months will be compared to our results from the final injection and three months to our follow-up results.

The Smith study [6] into the efficacy of PRP injections, demonstrated a reduction in total WOMAC scores from baseline over 12 months by 78% in comparison to a saline placebo which only reduced it by 7% [6]. This double-blind placebo-controlled RCT had clearly shown in comparison to the saline control that the three, weekly injections provided a conclusive statistically significant result. However, sample sizes were very small with 15 in each group and therefore studies with greater sample sizes are required. A similar double-blind RCT conducted by Patel et al. observed two different variations of administering PRP injections [19]. These were both single PRP injections with 10 times normal concentration and two PRP injections each given three weeks apart in comparison to saline placebo. Both groups showed reductions of baseline total WOMAC scores by 51% (single injection) and 43% (two injections), respectively in comparison to the placebo control which showed an increase of 20% over the six-month follow-up period [19]. Based on these studies a number of possible frequencies of administration can be utilized, but the use of three, weekly injections in the Smith study [6] demonstrated the greatest reduction in total WOMAC scores. Therefore, our practice was based on the technique and evidence demonstrated by the Smith study.

Pain scores reduced from 14.08 to 15.13% at the final injection followed by a 3.48% rise at follow-up. Our finding is in contrast to published literature. A double-blind RCT study performed by Cole et al. [16] demonstrated that PRP injections had no statistically significant difference in WOMAC pain scores in comparison to hyaluronic acid (HA) over the 12-month follow-up period. However, upon closer inspection, although no difference is shown between them, both therapies have shown a reduction in pain scores of 43.14% (PRP) and 33.51% (HA) from baseline reported in their data at 12 weeks with further reduction at 52 weeks [16]. The Smith study showed reduced pain scores of 79% in contrast to 28% in the placebo group at the three-month time point [6]. Likewise, Patel et al. further enforced the aforementioned results with a statistically significant reduction in pain scores [19].

In our study, stiffness and physical functions scores followed a very similar pattern with a reduction from 5.21 (stiffness) and 36.33 (function) at baseline, by 4.99% and 16.54% at the final injection and 6.72% and 4.84% at the follow-up six weeks after treatment. The Smith study in comparison demonstrated a 76% and 78% reduction in WOMAC stiffness and function scores, respectively, at three months. They further maintained this level until 12 months [6]. Patel et al. showed similar results with a reduction with a single IA injection [19]. They reported a reduction of 48% and 58% in stiffness and function, respectively, from baseline at three months. The maximum benefit was found to be at the three months follow-up with a decrease in efficacy at six months [19].

Total WOMAC scores showed reductions from 55.63 to 47.21 (-15.14%) before the last injection. At follow-up, the total WOMAC surprisingly showed a reduction to 54 (-2.93%) compared to baseline. The pattern of results with the reduction and rise as a whole was close to the baseline at the follow-up six weeks after treatment, which is well summarised by the total WOMAC scores, suggesting that the reduction is nothing more than a placebo effect that is maintained throughout the six to eight-week course of injections.

This is in contrast to the findings of the Smith study [6], where there was a 70% and 78% reduction from baseline (47) in total WOMAC scores over the two and three-month time periods the data was collected for [6]. In their study, BMI also ranged from 25±3 with a large majority not being overweight. A key explanation for this was that there was a much greater mix of individuals with a larger majority of these patients graded as having mild OA and not being as overweight in comparison to our study. In total, their study had 30 participants. Of these 30, 18 (60%) had mild with 12 (40%) having moderate OA. In our study 80% and 3.33% of the 31 patients had moderate and severe OA and only 16.7% had mild OA. The results from our study are therefore not entirely comparable. The greater proportion of moderate and severe OA patients in contrast to mild OA patients have very likely influenced the results demonstrating a classical placebo effect in contrast to the clear therapeutic benefit demonstrated in the Smith study [6].

As a result, it must be questioned whether PRP injections are beneficial for patients with grade 3 OA and whether they only be carried out in patients with grade 2 OA. Therefore, further studies could investigate this and make a clear distinction between both grades and determine if the same therapeutic benefit is gained in both categories. This theory is further enforced by the study published by Patel et al. [19]. Their double-blind RCT demonstrated a 60% reduction from baseline at the three-month point which is in keeping with very similar results to the Smith study [6,19]. A key difference, however, was this study used the Ahlback grading system with a large majority of participants in all groups having milder forms of OA grade 1 in comparison to moderate OA. In total, 74% and 75% of all patients included had this mild grade in both PRP treatment groups used [19]. Both studies are not entirely comparable with each other as a different classification of the OA system was utilized, however, this key variable must be further investigated based on results from this study.

We further separated our results into only mild OA patients as shown in Table 3. We did this to further determine if this group as evidenced and theorized, demonstrates a therapeutic benefit. From Table 3 we can see a similar trend in results with maximal benefit found before the third injection with all results increasing at the sixth-week follow-up after treatment. Our sample size of five patients, however, was extremely small and not significant enough to draw conclusions from.

Comments provided by patients after the course of treatment at follow-up showed that there was some benefit achieved, however, patients still experienced a lot of symptoms with little to no benefit achieved for the large majority. This could again be attributed to moderate OA patients (n=6 comments provided) likely having an advanced form of OA for this treatment to be of benefit and is in keeping with the aforementioned conclusions.

Limitations

A limitation of this study is that a large majority of the patients had moderate OA (80%) and results, therefore, were not comparable to the main RCT used as evidence for the practice. This study's limitations further include its retrospective nature and follow-up is limited to three months. A longer follow-up period such as six to 12 months may demonstrate differing results. This study only accounted for 32 patients under the care of two consultants and further studies with a larger sample size across multiple centers may provide higher level evidence for the practice.

## Conclusions

To conclude, based on the study conducted there appears to have been a placebo effect based on the results in all categories. There has been no statistically significant decrease in total WOMAC, pain, stiffness, and physical function scores from baseline up until follow-up six weeks after treatment. There were mixed comments provided by patients but the overarching theme was that the injections were beneficial for some people but did not completely provide symptomatic relief for any patient in this study. Though a clear trend has been demonstrated, further studies are needed with larger sample sizes and more significant subgroups based on weight and the Kellgren-Lawrence grade. As both mild and moderate OA patients receive PRP injections, further studies are needed to determine which grade of OA patients, grade 2 or 3, receives the greatest benefit.

## Figures and Tables

**Figure 1:**
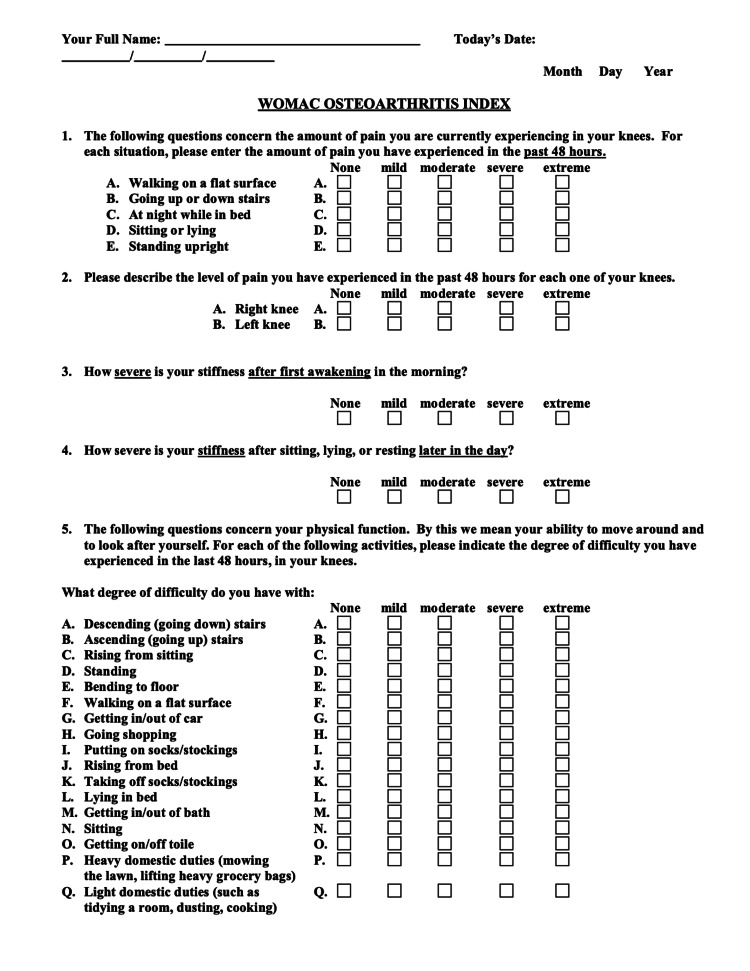
The Western Ontario and McMaster Universities arthritis index tool used to assess patients

## Appendices

Figure 1 shows the Western Ontario and McMaster Universities arthritis index (WOMAC) questionnaire used in this study to collect data on the modalities assessed. 
